# *CsLAC4*, regulated by *CsmiR397a*, confers drought tolerance to the tea plant by enhancing lignin biosynthesis

**DOI:** 10.1007/s44154-024-00199-1

**Published:** 2024-12-06

**Authors:** Hongbin Yang, Linxuan Xia, Jingshan Li, Xiaoyu Jia, Xinyue Jia, Yuying Qi, Youben Yu, Weidong Wang

**Affiliations:** College of Horticulture, Northwest A&F University, Yangling, Shaanxi 712100 China

**Keywords:** Tea plant, Drought, Laccase, MicroRNA, Lignin biosynthesis

## Abstract

Drought is a prevalent abiotic stress that commonly affects the quality and yield of tea. Although numerous studies have shown that lignin accumulation holds significant importance in conferring drought tolerance to tea plants, the underlying molecular regulatory mechanisms governing the tea plant's response to drought remain largely elusive. LACCASEs (LACs), which belong to the class of plant copper-containing polyphenol oxidases, have been widely reported to participate in lignin biosynthesis in plants and are implicated in numerous plant life processes, especially in the context of adverse conditions. In this study, we detected the upregulation of *CsLAC4* in response to drought induction. Remarkably, the overexpression of *CsLAC4* not only substantially increased the lignin content of transgenic *Arabidopsis thaliana* but also simulated the development of vascular tissues, consequently leading to a significant enhancement in drought tolerance. Moreover, via dual-luciferase assays and transient overexpression in tea leaves, we revealed that *CsLAC4* was negatively regulated by the upstream *CsmiR397a*. Interestingly, the expression of *CsmiR397a* was downregulated during drought stress in tea plants. *Arabidopsis thaliana* overexpressing *CsmiR397a* showed increased sensitivity to drought stress. By transient overexpression of *CsmiR397a* and *CsLAC4* in tea plant leaves, we verified that *CsLAC4*, which is regulated by *CsmiR397a*, conferred drought tolerance to tea plants by enhancing lignin biosynthesis. These findings enhance our understanding of the molecular regulatory mechanisms underlying the response of tea plants to drought stress.

**Supplementary Information:**

The online version contains supplementary material available at 10.1007/s44154-024-00199-1.

## Introduction

The tea plant [*Camellia sinensis* (L.) O. Kuntze] is the most popular non-alcoholic beverage crop in the world and is loved for its unique flavor and numerous nutritional benefits (Li et al. 2022b). However, drought is one of the major abiotic stresses faced by tea plantations in China (Zhang et al. 2023). Indeed, drought stress can severely hinder the growth of tea plants and even result in leaf damage, which in turn affects the tea yield (Zhang et al. 2022b). Additionally, drought leads to a significant reduction in important flavor compounds in tea leaves, including catechins, caffeine, theanine and certain free amino acids, thereby reducing tea quality (Wang et al. 2016; Shen et al. 2022). Therefore, understanding of the drought-stress response mechanism of tea plants is crucial for improving tea yield and quality.

Numerous studies have shown that plant responses to drought stress are often accompanied by lignin accumulation (Xie et al. 2024), which occurs predominantly in xylem cells and xylem fibers (Liu et al. 2021). Drought stress damages xylem tracheal elements (TEs) that are responsible for water and inorganic salt transport, resulting in the disruption of plant hydraulic conductivity (Choi et al. 2023). However, lignin accumulation may enhance the tensile strength of TEs and reduce drought damage, thereby increasing plant drought tolerance (Ménard et al. 2022). LACCASEs (LACs), such as multicopper oxidase, have been widely reported to be involved in lignin biosynthesis in plants (Tang et al. 2023), which in turn plays an important role in a range of life processes in plants, particularly in response to adverse conditions (Janusz et al. 2020). For example, *ZmLAC3/9* promotes lignin deposition in maize vascular bundles, thereby increasing their tolerance to high N and salinity stress (Sun et al. 2018; Qin et al. 2023). Similarly, *CsiLAC18* promotes lignin accumulation and affects cold tolerance in citrus (Xu et al. 2022). Recently, LACs have been reported to be involved in the response to drought stress. For example, six LACs may play roles in lignin accumulation in chickpea roots under drought stress (Sharma et al. 2020). Meanwhile, *PeuLAC2* thickens the cell wall and improves water transport to enhance drought tolerance in *Populus euphratica* (Niu et al. 2021). Consistently, in tea plants, laccase family genes have been identified and their potential involvement in processes such as young shoot development, hormonal responses, and defense against phytophagous insects has been revealed by expression level analysis (Yu et al. 2021). However, the precise biological functions that *CsLACs* perform in response to drought stress remain unclear.

The regulatory effects of *miR397* on *LACs* have been widely reported in recent years, and they have been implicated in plant growth and development as well as stress responses (Huang et al. 2021). Thus, for example, in *Arabidopsis thaliana*, the *miR397b*-*LAC2* module influences tolerance to water deficit and cadmium stress by regulating root lignification (Gaddam et al. 2024; Khandal et al. 2020). Similarly, the *GhmiR397*-*GhLAC4* module mediates lignin accumulation, which improves cotton tolerance to *V. dahlia* (Wei et al. 2021). Furthermore, *CamiR397* targets *LAC4* and *LAC17*, which affect chickpea tolerance to drought and dry root rot (Sharma et al. 2023). Recently, bioinformatic studies have predicted that *CsLACs* may be regulated by *CsmiR397a*, which is involved in tea plants response to gray blight (Zhu et al. 2023). Similarly, we have previously confirmed that the *CsmiR397a*-*CsLAC17* module play a role in balancing tenderness and gray blight resistance in young tea shoots (Yang et al. 2024). However, little is known about the role and regulatory relationships of *CsmiR397a* targeting *CsLACs* in response to drought stress.

Our previous study identified a LAC gene (*CsLAC4*, gene ID: *CSS0015036*) that was significantly induced under drought stress (Wang. 2016), and we speculated that it might play an important role in the response to drought stress in tea plants. In this study, we investigated the expression levels of *CsLAC4* in response to drought stress, and the biological function of *CsLAC4* under drought stress by heterologous expression in *Arabidopsis thaliana* and native expression in tea plants. Meanwhile, the regulatory effects of *CsmiR397a* on *CsLAC4* and the underlying synergistic mechanisms of action in response to drought stress were also validated. Our study contributes to the understanding of the molecular regulatory mechanisms of tea plants in response to drought and provides strategies and insights for the selection of drought-tolerant tea plant cultivars.

## Results

### *CsLAC4* expression and lignin accumulation in roots were induced under drought stress

**Fig. 1:**
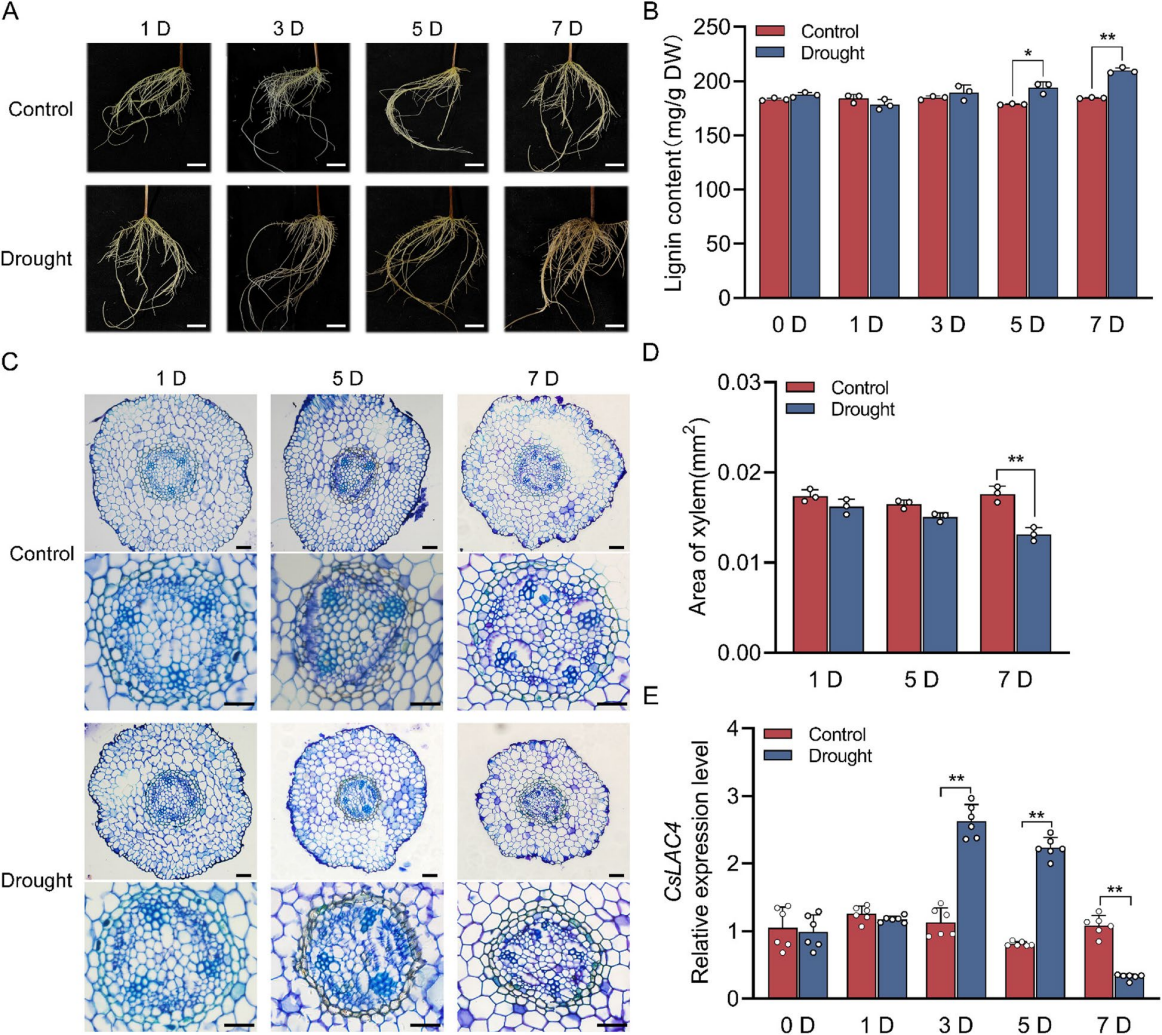
*CsLAC4* expression and lignin accumulation are induced in roots under drought stress. **A** Phenotypes of tea plant roots under drought treatment. Scale bars, 30 cm. **B** Lignin content of tea plant roots under drought treatment (*n* = 3). **C** Toluidine blue staining of tea plant roots sections under drought treatment. Scale bars, 100 μm. **D** Area of xylem in tea plant roots under drought treatment (*n* = 3). **E** Expression levels of *CsLAC4* in roots under drought treatment (*n* = 6). Each bar indicates the mean ± SD. Asterisks indicate significant differences relative to the control, *p < 0.05, **p < 0.01

Tea plant roots showed gradual browning under drought stress compared to the controls (Fig. 1A), concomitant with a significant accumulation of lignin, especially at 5 and 7 d after drought treatment initiation (Fig. 1B). Histochemical staining showed a reduction in root xylem area under drought stress, which was accompanied by a significant increase in lignin deposition (Fig. 1C and D). Furthermore, the expression level of *CsLAC4* in the roots initially increased, followed by a decrease in response to drought stress (Fig. 1E). These findings indicated that *CsLAC4* may be involved in the accumulation of root lignin under drought stress.

### Sequence analysis and subcellular localization of the CsLAC4 protein

**Fig. 2:**
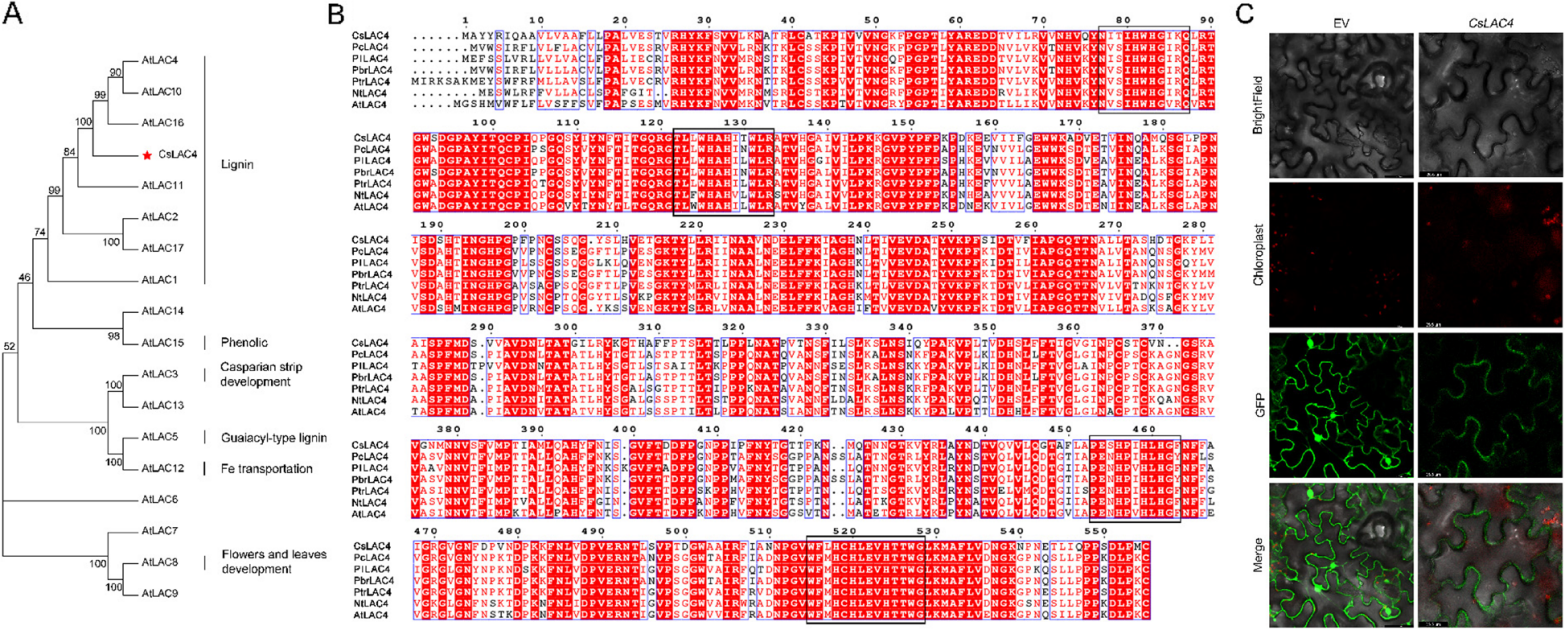
Sequence analysis and subcellular localization of CsLAC4 protein. **A** Phylogenetic analysis of CsLAC4 and 17 Arabidopsis laccases. **B** Alignment was conducted between the CsLAC4 protein and other laccases (AtLAC4, PbrLAC4, PtrLAC4, PlLAC4 and NtLAC4) sequences. The copper-binding domains are marked with a black box. **C** The subcellular localization of CsLAC4

Phylogenetic analysis showed that CsLAC4 clustered with LACs involved in lignin biosynthesis and had the highest homology with AtLAC4 (Fig. 2A). Sequence comparison analyses showed that the protein sequence of CsLAC4 was highly similar to that of LAC4 from other species and contained four conserved copper-binding structural domains, making it a typical member of the laccase family (Fig. 2B). Moreover, the result of subcellular localization indicated that CsLAC4 might be localized to the cell wall (Fig. 2C). These results indirectly suggest the potential involvement of CsLAC4 in lignin biosynthesis within cell walls.

### Overexpression of *CsLAC4* conferred drought tolerance to transgenic *Arabidopsisthaliana*

**Fig. 3:**
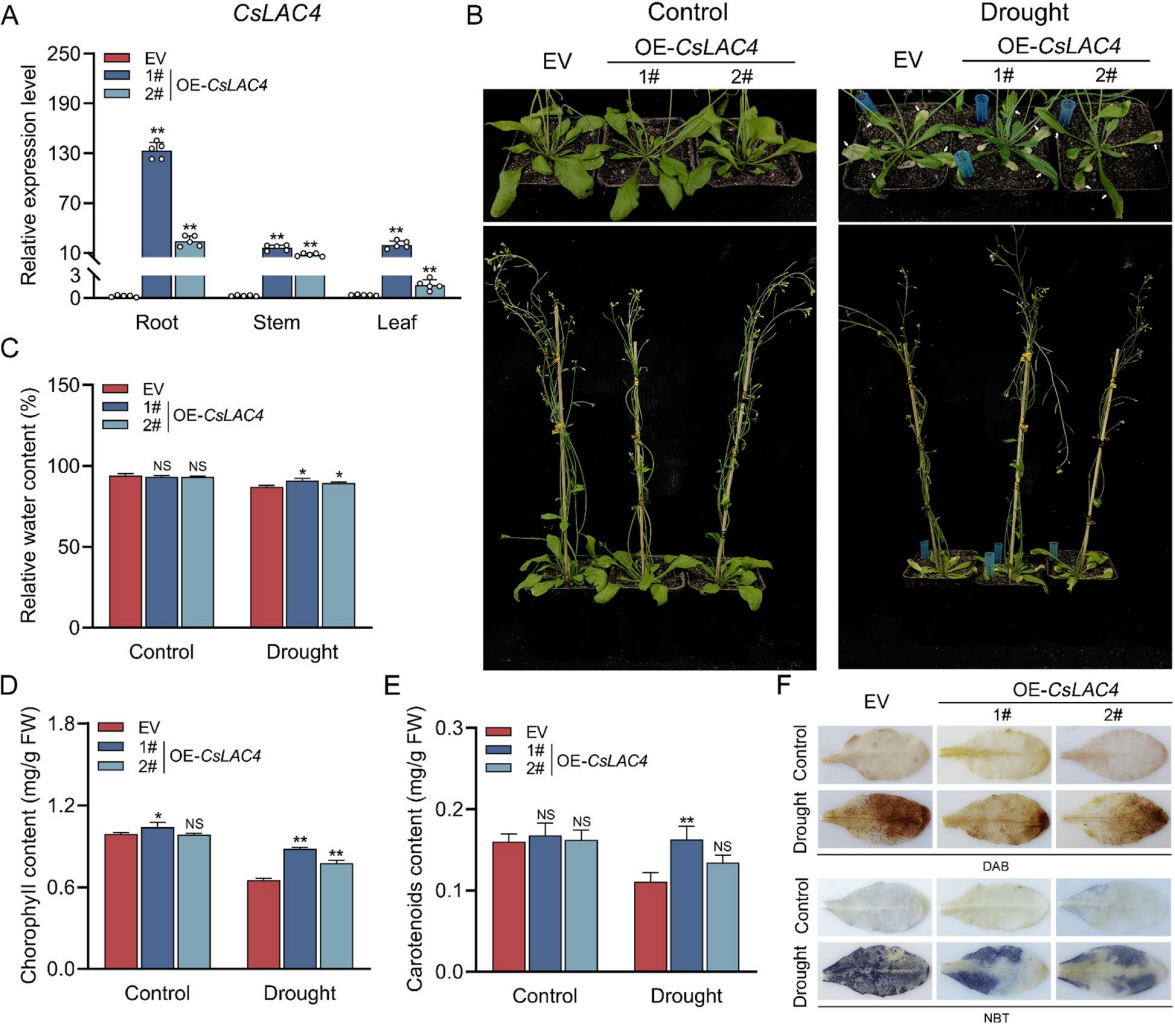
Overexpression of *CsLAC4* confers drought tolerance in transgenic *Arabidopsis thaliana*. **A** Expression levels of *CsLAC4* in two transgenic Arabidopsis lines (*n* = 5). **B** Phenotypes of OE-*CsLAC4* Arabidopsis under drought condition, water-loss wilting and yellowing leaves have been pointed out with white arrows. **C-E** Determination of relative water content and photosynthetic pigment content in OE-*CsLAC4* Arabidopsis (*n* = 3). **F** ROS staining in OE-*CsLAC4* Arabidopsis under drought treatment (*n* = 3). Each bar indicates the mean ± SD. Asterisks indicate significant differences relative to the EV, *p < 0.05, **p < 0.01

The 35 s::*CsLAC4*::GFP vector was constructed (Fig. S1A) and two homozygous overexpressing *CsLAC4* (OE-*CsLAC4*) lines were obtained (Fig. S1B and C). Subsequently, the results of qRT-PCR showed that *CsLAC4* was highly expressed in different tissues of the two overexpressing lines, particularly in the roots (Fig. 3A). In response to drought conditions, OE-*CsLAC4* seedlings displayed less severe yellowing and enhanced root development, along with an increased level of drought tolerance compared to seedlings transferred to the empty vector (EV) (Fig. S2). Similarly, four-week-old OE-*CsLAC4* Arabidopsis with less water-loss wilting and yellowing showed enhanced drought tolerance (Fig. 3B). Specifically, OE-*CsLAC4* plants exhibited higher levels of water, chlorophyll and carotenoid content (Fig. 3C-E). Furthermore, DAB and NBT staining demonstrated that the accumulation of H₂O₂ and O₂^−^ under drought stress was significantly lower in OE-*CsLAC4* plants than in EV plants (Fig. 3F). These results indicate that overexpression of *CsLAC4* may enhance drought tolerance in transgenic *Arabidopsis thaliana*.

### Overexpression of *CsLAC4* promoted lignin accumulation and xylem development in transgenic *Arabidopsisthaliana*

**Fig. 4:**
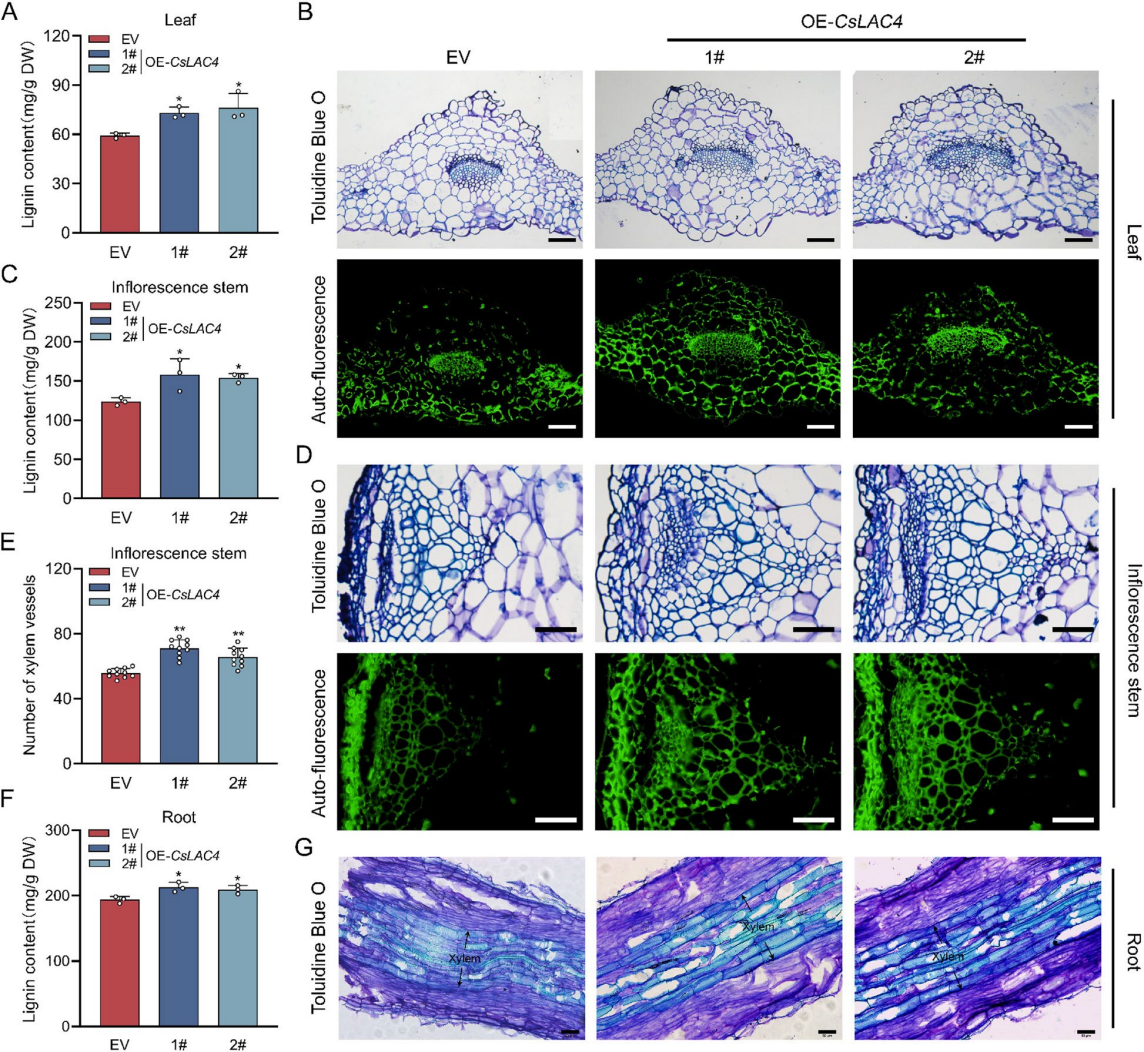
Overexpression of *CsLAC4* promotes lignin content and xylem development in transgenic *Arabidopsis thaliana*. **A** Leaf lignin content in OE-*CsLAC4* Arabidopsis (*n* = 3). **B** Toluidine blue staining and lignin autofluorescence in leaves. Scale bars, 100 μm. **C** Lignin content of inflorescence stem (*n* = 3). **D** Toluidine blue staining and lignin autofluorescence in inflorescence stem. Scale bars, 50 μm. **E** Number of xylem vessels in inflorescence stem (*n* = 10). **F** Toluidine blue staining in root. Scale bars, 50 μm. Each bar indicates the mean ± SD. Asterisks indicate significant differences relative to the EV, *p < 0.05, **p < 0.01

As shown in Fig. 4A, lignin content in the leaves of two OE-*CsLAC4* lines was approximately 23% and 28% higher than that in the leaves of EV plants, respectively. Histochemical staining revealed increased xylem TEs in the leaves of OE-*CsLAC4* plants (Fig. 4B). Similarly, lignin content and xylem TE number were higher in the inflorescence stems of OE-*CsLAC4* plants than in those of EV plants (Fig. 4C-E). Furthermore, the lignin content of the roots in OE-*CsLAC4* plants was significantly higher than that of the EV plants (Fig. 4F). Longitudinal sections of the roots showed that the xylem width of OE-*CsLAC4* roots was greater than that of the EV plants (Fig. 4G). In view of these results, we speculated that the overexpression of *CsLAC4* increased lignin content and promoted vascular bundle development in Arabidopsis, which may be associated with the enhanced drought tolerance observed.

### *CsLAC4* expression was targeted and regulated by *CsmiR397a*

**Fig. 5:**
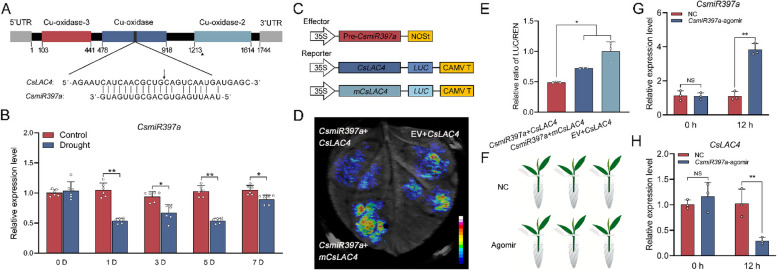
*CsLAC4* expression is targeted and regulated by *CsmiR397a*. **A** Predication of target binding sequences of *CsLAC4* matching *CsmiR397a. ***B** Expression levels of *CsmiR397a* in tea plant roots under drought treatment (*n* = 6). **C** Schematic representation of effectors and reporters used for the dual-luciferase assay. **D** Results of the dual luciferase imaging. **E** Determination of relative activity of LUC (*n* = 3). **F** Schematic diagram of young tea shoots overexpressing *CsmiR397a*. **G-H** Expression levels of *CsmiR397a* and *CsLAC4* in young tea shoots overexpressing *CsmiR397a* (*n* = 3). Each bar indicates the mean ± SD. *p < 0.05, **p < 0.01

Bioinformatic predictions showed that the CDS of *CsLAC4* has the target sequence matching *CsmiR397a* and is located in the Cu-oxidation structural domain (Fig. 5A). The results of qRT-PCR demonstrated that *CsmiR397a* expression was repressed in response to drought conditions (Fig. 5B). The vector used for the dual-luciferase experiment was subsequently constructed (Fig. 5C), and the results of tobacco co-transformation showed that the fluorescence intensity of co-expressed *CsmiR397a* and *CsLAC4* was significantly lower than that of the control (co-expression of EV and *CsLAC4*), but the fluorescence intensity of co-expressed *CsmiR397a* with m*CsLAC4* (with the GC of the 10th and 11th bases replaced by AA) was similar (Fig. 5D). Furthermore, LUC activity analysis showed that the co-expression of *CsmiR397a* and *CsLAC4* was significantly lower than control and co-expression of m*CsLAC4* (Fig. 5E). In addition, we used *CsmiR397a*-agomir to overexpress *CsmiR397a* in tea plants (Fig. 5F), and the results showed that the expression level of *CsmiR397a* significantly increased after 12 h of incubation (Fig. 5G), whereas the expression level of *CsLAC4* significantly decreased (Fig. 5H). Based on these results, we inferred that *CsmiR397a* might play a role in the response of tea plants to drought stress by affecting lignin accumulation through the regulation of *CsLAC4*.

### Overexpression of *CsmiR397a* reduced drought tolerance in transgenic *Arabidopsisthaliana*

**Fig. 6:**
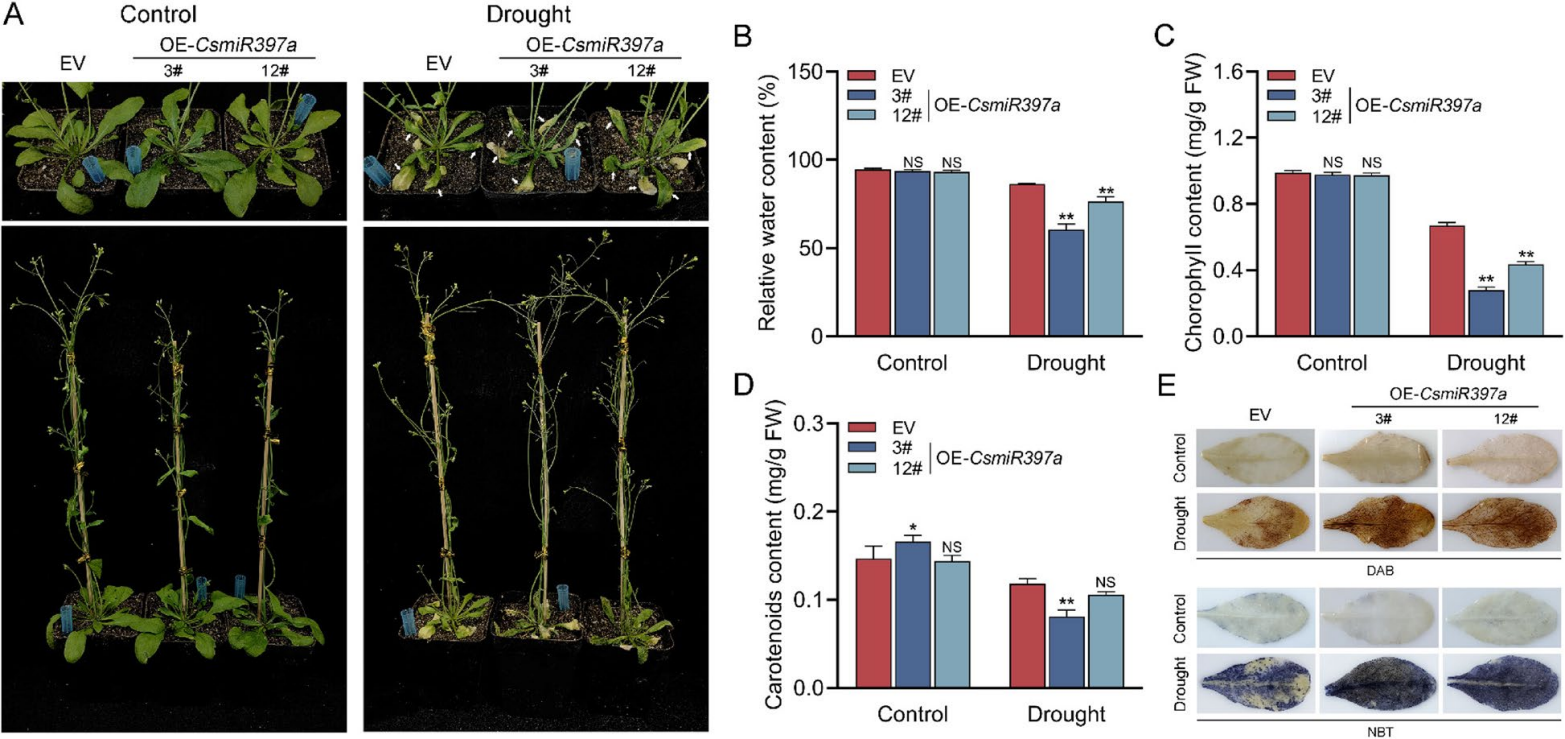
Overexpression of *CsmiR397a* reduces drought tolerance in transgenic *Arabidopsis thaliana*. **A** Phenotypes of OE-*CsmiR397a* Arabidopsis under the drought condition, water-loss wilting and yellowing leaves have been pointed out with white arrows. **B-D** Determination of relative water content and photosynthetic pigment content in OE-*CsmiR397a* Arabidopsis (*n* = 3). **E** ROS staining in OE-*CsLAC4* Arabidopsis under drought treatment (*n* = 3). Each bar indicates the mean ± SD. Asterisks indicate significant differences from the EV, *p < 0.05, **p < 0.01

Compared with EV seedlings, OE-*CsmiR397a* seedlings showed more pronounced yellowing, greater inhibition of root growth, and reduced drought tolerance under drought stress (Fig. S4). Indeed, four-week-old OE-*CsmiR397a* Arabidopsis showed more severe wilting and yellowing due to water loss and exhibited reduced drought tolerance (Fig. 6A). Specifically, OE-*CsmiR397a* plants had lower water, chlorophyll and carotenoid contents (Fig. 6B-D). Furthermore, DAB and NBT staining showed that the accumulation of H₂O₂ and O₂^−^ was significantly higher in OE-*CsmiR397a* plants than in EV plants under drought stress (Fig. 6E). In addition, the expression of *AtLAC4*, a homolog of *CsLAC4*, was significantly suppressed in OE-*CsmiR397a* plants (Fig. S4). These findings indicate that *CsmiR397a* may have reduced drought tolerance in transgenic Arabidopsis by suppressing *AtLAC4* expression.

### *CsLAC4* regulated by *CsmiR397a* influence lignin biosynthesis to improve drought tolerance of tea plants

**Fig. 7:**
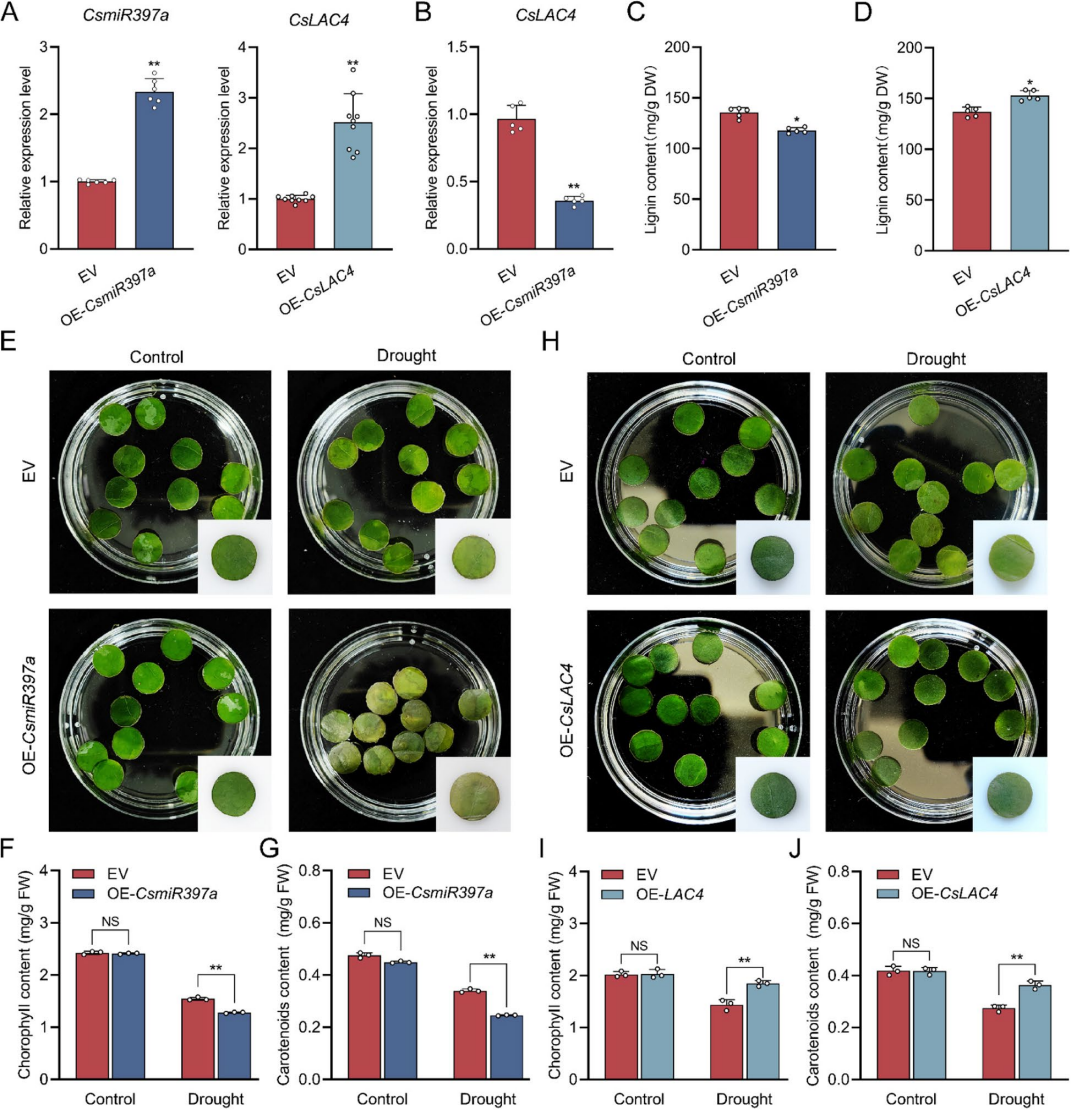
*CsLAC4* regulated by *CsmiR397a* influence lignin biosynthesis to improve the tea plant drought tolerance. **A** Transient overexpression of *CsmiR397a* and *CsLAC4* in tea leaves (*n* ≥ 5). **B** The expression level of *CsLAC4* in OE-*CsmiR397a* leaves (*n* = 5). **C** Lignin content in OE-*CsmiR397a* leaves (*n* = 5) **D** Lignin content of leaves overexpressing *CsLAC4* (*n* = 5). **E–H** Phenotypes of leaves overexpressing *CsmiR397a* and *CsLAC4* under drought stress (*n* = 5). **F-J** Chlorophyll and carotenoid content of *OE-CsmiR397a* and *OE-CsLAC4* leaves under drought stress (*n* = 3). Each bar indicates the mean ± SD. Asterisks indicate significant differences relative to the EV, *p < 0.05, **p < 0.01

Transient overexpression of *CsmiR397a* and *CsLAC4* in tea plant leaves was confirmed by qRT-PCR analysis and GFP fluorescence signal detection (Fig. 7A and S5). The expression of *CsLAC4* was reduced by 64% in OE-*CsmiR397a* leaves (Fig. 7B). Meanwhile, the lignin content was reduced by approximately 13% in OE-*CsmiR397a* leaves compared to that in EV leaves (Fig. 7C). Conversely, lignin content of the leaves overexpressing *CsLAC4* increased by approximately 10% (Fig. 7D). Moreover, OE-*CsmiR397a* leaf discs suffered greater damage and more severe yellowing under drought conditions than the EV leaf discs (Fig. 7E). Additionally, results of photosynthetic pigment content showed that OE-*CsmiR397a* leaf discs showed reduced levels of chlorophyll and carotenoids in comparison to EV leaf discs under drought stress (Fig. 7F-G). In contrast, leaf discs overexpressing *CsLAC4* showed a slight loss of greening and better drought tolerance (Fig. 7H). Meanwhile, chlorophyll and carotenoid contents of OE-*CsLAC4* leaf discs were significantly higher under drought stress (Fig. 7I-J). These findings indicate that *CsLAC4* was regulated by *CsmiR397a*, thereby influencing lignin biosynthesis, which is crucial for enhancing drought tolerance in tea plants.

## Discussion

Drought is an important abiotic stress factor with detrimental effects on plant growth and development. In the case of tea plants, drought stress affects not only the quality of tea leaves, but also their survival (Shao et al. 2023). Recently, there has been considerable interest in the mechanisms by which plants respond to drought stress, among which lignin accumulation has been confirmed as a key factor in enhancing plant drought resistance (Hou et al. 2022), as is reportedly the case in rice and poplar root systems, which show a higher degree of lignification under drought stress (Bang et al. 2022; Zhou et al. 2020). Our findings indicate that accumulation of lignin in the roots of tea plants subjected to drought stress may be beneficial for maintaining the stability of water transport. This indicates that lignin accumulation is essential for enhancing drought tolerance in tea plants, which is consistent with the findings of Li et al. (2024) and Geng et al. (2018). To date, it has been demonstrated that a large number of lignin biosynthesis-related genes are involved in plant response to drought. For example, *MeRAV5* promotes *MeCAD* expression, thereby regulating lignin accumulation in cassava and enhancing its drought tolerance (Yan et al. 2021). Similarly, *PoCCoAOMT* promotes lignin biosynthesis, which in turn confers drought tolerance to peony (Zhao et al. 2021). Meanwhile, *PeLAC10* confers tolerance to drought in bamboo by participating in lignin biosynthesis (Li et al. 2020). Consistently, *CaLAC4*, a homologue of *AtLAC4*, has also been implicated in lignin biosynthesis in chickpea under drought stress (Sharma et al. 2023). Here, we found that *CsLAC4* expression in tea plant roots showed a notable increase in response to drought stress. Additionally, phylogenetic analysis showed that CsLAC4 clustered into a branch with LACs that were confirmed to be involved in lignin biosynthesis in Arabidopsis (Blaschek et al. 2023). A substantial body of evidence indicates that the cell wall, which is the site of the late stages of lignin biosynthesis is the main site where laccase is located. For example, both PcLAC4 and CsiLAC4 are localized in the cell wall and are involved in lignin biosynthesis in pears and citrus, respectively (Yang et al. 2023; Huang et al. 2022). Similarly, the CsLAC4 protein was also localized to the cell wall. Based on these findings, we hypothesized that *CsLAC4* may play an important role in lignin accumulation under drought stress. This finding was further confirmed by a significant increase in lignin content observed in transgenic Arabidopsis overexpressing *CsLAC4*. Enhanced lignin biosynthesis facilitates vascular tissue maturation and development, which is crucial for optimizing water transport (Li et al. 2022a, 2023). Interestingly, roots, leaves and inflorescence stems of overexpressing *CsLAC4* Arabidopsis showed enhanced vascular tissue development, improved water transport capacity, photosynthetic pigment content and ROS scavenging capacity, further reducing the sensitivity to drought. Similarly, transient overexpression of *CsLAC4* significantly increased lignin content in tea leaves, thereby improving drought tolerance. These results suggested that *CsLAC4* promoted lignin accumulation and vascular tissue development to improve drought tolerance in tea plants.

Further, *MiR397* regulates multiple *LACs,* affecting cell wall lignification and multiple life processes in plants. For example, *KomiR397* affects lignification and cadmium tolerance in the root epidermis of *Kandelia obovata* by regulating the expression of *KoLAC4/7/17* (Pan et al. 2024). Additionally, *MrLAC17*, which is regulated by *MrmiR397a*, enhances lignin synthesis in alfalfa, leading to an erect stem phenotype (Zhang et al. 2022a). Furthermore, a study on the tea plant have predicted that *miR397* may target *LACs* in response to infection by *Pestalotiopsis*-like species (Zhou et al. 2022). In a previous study, we demonstrated that the *CsmiR397a*-*CsLAC17* module plays a role in maintaining the balance between tenderness and gray blight resistance in young tea shoots (Yang et al. 2024). Interestingly, in this study, the expression of *CsmiR397a* was suppressed by drought stress and negative correlated with the expression level of *CsLAC4*, suggesting that *CsmiR397a* is synergistically involved with *CsLAC4* in the drought response of the tea plant. In contrast, *CsmiR397a*-overexpressing Arabidopsis showed a weaker capacity for water transport, photosynthetic pigment accumulation, and ROS scavenging capacity under drought stress. As found in our previous study, overexpression of *CsmiR397a* led to reduced lignin content and poor development of vascular tissues in Arabidopsis, which in turn led to impaired water transport and reduced drought tolerance (Yang et al. 2024). Notably, the expression of *AtLAC4*, which is most homologous to *CsLAC4*, was also significantly reduced in *CsmiR397a*-overexpressing Arabidopsis. The regulatory relationship between *CsmiR397a* and *CsLAC4* in the tea plant was further confirmed using miRNA-agomir and transient overexpression technologies. Specifically, the overexpression of *CsmiR397a* resulted in the inhibition of *CsLAC4* accumulation, which in turn led to a reduction in lignin content and increased the sensitivity of the tea plant to drought. These findings indicate that *CsmiR397a* influenced lignin accumulation by regulating the expression of *CsLAC4*, which in turn affected the level of drought tolerance of tea plants.

## Conclusion

**Fig. 8:**
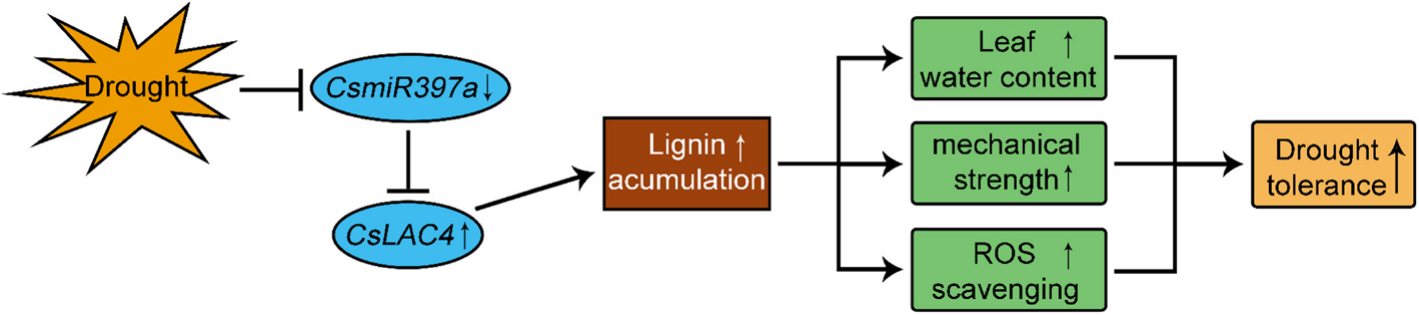
*CsLAC4*, regulated by *CsmiR397a*, confers drought tolerance by increasing lignin biosynthesis in the tea plant. Drought stress-induced downregulation of *CsmiR397a* enhances the expression of *CsLAC4*, which leads to the lignin accumulation, promotes water transport, mechanical support and ROS scavenging, thereby conferring drought tolerance to the tea plant

In summary, this study investigated *CsmiR397a* regulated *CsLAC4* by affecting lignin biosynthesis and played a significant role in the development of drought resistance in tea plants. Specifically, drought stress inhibited the expression of *CsmiR397a* leading to a high expression of *CsLAC4*, which in turn enhanced lignin accumulation and improved the stability of vascular tissues, which ensured water transport, maintained normal photosynthesis and ROS scavenging activity, and improved the level of drought tolerance of tea plants (Fig. 8). Our study clarified the biological function of *CsLAC4* and its regulatory mechanism under drought stress. These findings are expected to provide new insights into the enhancement of the tea plant tolerance to drought, tea yield and quality.

## Materials and methods

### Plant materials and treatments

Annual tea plant cuttings (*C. sinensis* cv. ‘Zhongcha 108’) were precultured hydroponically in a greenhouse at Northwest A&F University as previously described (Cheng et al. 2021). The nutrient solution containing 10% PEG 6000 was used to simulate drought stress. After 0, 1, 3, 5, and 7 days (d) of incubation under normal and drought conditions, respectively, tea plant roots were collected and snap-frozen in liquid nitrogen and stored at −80 °C. *Arabidopsis thaliana* was grown as previously described (Xiao et al. 2023). Briefly, the seeds were cultured in 1/2 MS solid medium for seven days. Then, some of the seedlings were transferred to 1/2MS medium containing 300 mM mannitol for 5 or 7 d to simulate drought stress, the number of plants used for per treatment for each line exceeded 15. The remaining seedlings were transferred to pots, cultured for three weeks, and then watered with 20% PEG 6000 to simulate drought treatment, the number of plants used for per treatment for each line exceeded 30.

### Cloning of *CsLAC4 *and *Arabidopsis* plant transformation

The CDS sequence of *CsLAC4* was cloned using specific primers (Table S1). Subsequently, the sequence was incorporated into the binary vector pCambia2300-GFP, resulting in 35S::*CsLAC4*::GFP vector construction. The obtained vector was transformed into wild-type Arabidopsis using the flower-dipping method with *A. tumefaciens* (GV3101). Subsequently, T2 and T3 homozygous lines were used for the functional validation of *CsLAC4*.

### qRT-PCR analysis

RNA was extracted using the CTAB method (Bai et al. 2023) and cDNA was synthesized was performed using the miRNA 1st strand cDNA synthesis kit (Accurate, China). Subsequently, qRT-PCR was performed using LightCycler 480 (Roche, Basel, Switzerland). Relative gene expression was then calculated using the 2^−ΔΔCt^ method. *CsActin* and *CsmiR222* were used as the internal reference genes for *CsLAC4* and *CsmiR397a*, respectively. Three biological replicates were used to analyze each gene. All primers used are listed in Table S1.

### Subcellular localization analysis

The 35S::*CsLAC4*::GFP vector and the EV obtained above were transiently transformed into *N. benthamiana* leaf blades using *A. tumefaciens* (GV3101). After 48 h, transformed leaves were collected to detect the subcellular localization of the CsLAC4 protein using laser confocal microscopy, as previously described (Zhang et al. 2021).

### Dual-luciferase reporter assay

For this experiment, 35S::*CsmiR397a*, 35 s::*CsLAC4*::LUC and 35 s::*mCsLAC4*::LUC vectors were constructed. The mutant sequence, *mCsLAC4*, is a change of two bases (GC to AA) at the cleavage site of the target sequence. *A. tumefaciens* (GV3101 psoup) containing the above vectors was co-transformed into *N. benthamiana* leaves. Observations were made by CCD (Princeton, USA) after a 2d period of infiltration. The activities of LUC and REN were determined using the Dual-Luciferase Reporter Kit (Transgen, China).

### Specific overexpression of *CsmiR397a*

To specifically overexpress *CsmiR397a* in tea plants, the previously described RNA oligonucleotide technique was used with slight modifications (Zhou et al. 2024). Specifically, young shoots of the tea plant (one bud and two leaves) were collected and placed in nuclease-free water containing 20 μM *CsmiR397a*-agomir (*CsmiR397a* overexpression) or negative control. After a 12-h period of incubation, leaf samples were collected for qRT-PCR analysis. The sequences of the RNA oligonucleotides are listed in Table S1.

### Transient overexpression of *CsmiR397a* and *CsLAC4* in tea leaves and leaf discs assay

A slightly modified version of the previously reported method was used. *A. tumefaciens* (GV3101 pSoup-19) containing the 35 s::*CsmiR397a* and 35 s::*CsLAC4*::GFP vectors was cultivated in LB medium and was resuspend to OD_600_ of 1.0 in the injection solution (25 mM MES, 150 μM acetosyringone, 2 mM Na_3_PO_4_, and 0.5% (m/v) D-glucose). *A. tumefaciens* containing the 35 s::GFP vector served as the control. Three days after injection, leaves were harvested for gene expression analysis and lignin content determination.

To validate the functionality of *CsmiR397a* and *CsLAC4* in conferring drought tolerance in the tea plant, two discs (14 mm in diameter) were cut from each leaf, injected with *A. tumefaciens* containing the above vectors, and incubated for 5 d at 25℃ in 15 ml distilled water as the control or in solutions containing 20% PEG 6000 as drought stress. After cutting, the remaining tea plant tissues were collected for qRT-PCR identification, and the identified positive plants were used for phenotypic observation and determination of photosynthetic pigment content.

### Determination of lignin content

Inflorescence stems, rosette leaves, and roots of four-week-old transgenic Arabidopsis were collected and stored at −80℃. Lignin content was determined according to the acetobromine method described previously (Wang et al. 2024). Three independent biological replicates were included and samples in each biological replicate were obtained from seven different plants.

### Histochemical staining analysis

Four-week-old Arabidopsis leaves, inflorescence stems, and roots were fixed in FAA fixative (formaldehyde: glacial acetic acid:50% alcohol = 1:1:17) for 48 h. Sections with a thickness of 10 μm were produced using an RM2235 microtome (Leica, Germany) after embedding in paraffin. Then, these sections were stained with 1% toluidine blue solution. A portion of the stained samples was observed in bright field and another portion of the unstained samples was used to observe lignin autofluorescence under UV light using an Olympus BX-53 microscope (Olympus, Japan).

### Determination of relative water content and photosynthetic pigment content

Leaf fresh weight was recorded as M1 and dry weight was recorded as M2. The relative water content was calculated using the following formula: (M1-M2)/M1. Photosynthetic pigment content was determined according to a previously described method (Qin et al. 2024). Three independent biological replicates were included and samples in each biological replicate were obtained from seven different plants.

### ROS staining analysis

DAB staining dry powder of appropriate quality was diluted to 1 mg/mL in distilled water, and concentrated hydrochloric acid was added dropwise to a pH of 3.8. Prior to utilization, the pH of the staining solution was adjusted to 5.8 with sodium hydroxide. Plant tissues were immersed in the DAB staining solution and incubated at 28℃ in the dark for 8 h. Subsequently, the solution was decolorized using 85% ethanol.

NBT staining: Dry NBT powder was dissolved in 10 mM potassium phosphate buffer (pH = 7.6) and diluted to 0.5 mg/mL. Plant tissues were immersed in the NBT staining solution and incubated at 28℃ for 3 h before distaining with 85% ethanol.

### Statistical analysis

Statistical analysis of the data was conducted using GraphPad Prism 9.0 software. Student’s *t*-tests were used to assess the significance of the differences between two samples, whereas one-way analysis of variance was used for the remaining data. Values shown in tables and figures are means ± standard deviation.

## Supplementary Information

Additional file 1: Fig. S1. Identification of Arabidopsis overexpressing *CsLAC4*. Fig. S2. Validation of drought tolerance in OE-*CsLAC4* Arabidopsis. Fig. S3. Validation of the regulation of *AtLAC4* by *CsmiR397a* in Arabidopsis. Fig. S4. Validation of drought tolerance in OE-*CsmiR397a* Arabidopsis. Fig. S5. EV, CsLAC4-GFP fusion protein in bright field and GFP field of fluorescence microscopy.

Additional file 2: Table S1 Primers used in this study.

## Data Availability

All data and materials included in this study are available upon request by contact with the corresponding author.

## Abbreviations

*V. dahlia*: *Verticillium dahlia*
*A. tumefaciens*: *Agrobacterium tumefaciens*
*N. benthamiana*: *Nicotiana benthamiana*
MiRNA: MicroRNA
LAC: Laccase
CAD: Cinnamyl alcohol dehydrogenase
CCoAOMT: Caffeoyl-CoA-3-O-methyltransferase
CDS: Coding sequence
ROS: Reactive oxygen species
TEs: Tracheal elements
MS: Murashige and skoog
PEG: Polyethyleneglycol
FAA: Formaldehyde glacial acetic acid alcohol
DAB: Diaminobenzidine
NBT: Nitro blue tetrazolium
GFP: Green fluorescent protein
CTAB: Cetyltrimethylammonium Bromide
cDNA: complementary DNA
qRT-PCR: Quantitative real-time polymerase chain reactions
