# Supplementary material for: CsLAC4, regulated by CsmiR397a, confers drought tolerance to the tea plant by enhancing lignin biosynthesis
Source: Stress Biol. 2024 Dec 6;4(1):50. doi: 10.1007/s44154-024-00199-1 (PMC11624182; doi:10.1007/s44154-024-00199-1)
Supplement: Supplementary file 1 — Additional file 1: Fig. S1. Identification of Arabidopsis overexpressing CsLAC4. Fig. S2. Validation of drought tolerance in OE-CsLAC4 Arabidopsis. Fig. S3. Validation of the regulation of AtLAC4 by CsmiR397a in Arabidopsis. Fig. S4. Validation of drought tolerance in OE-CsmiR397a Arabidopsis. Fig. S5. EV, CsLAC4-GFP fusion protein in bright field and GFP field of fluorescence microscopy. [file 44154_2024_199_MOESM1_ESM.docx]

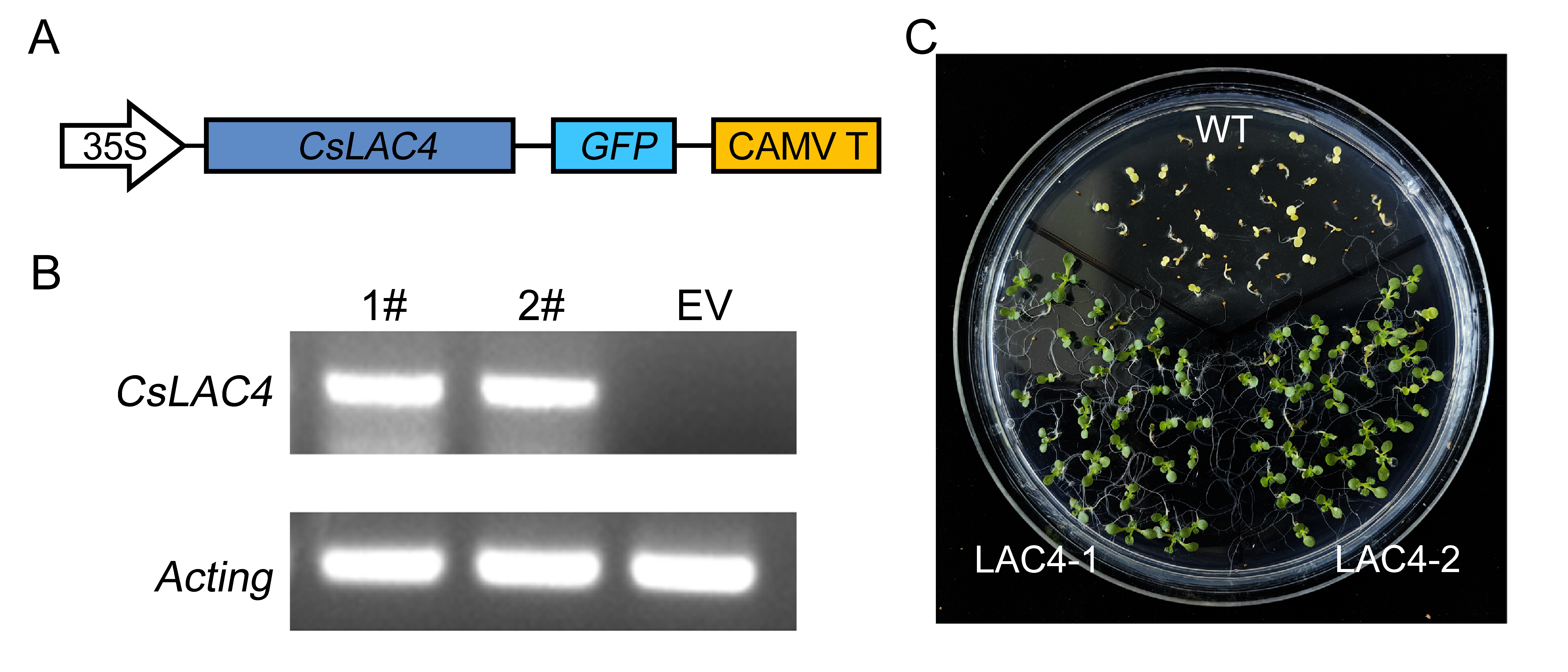


**Fig. S1** Identification of Arabidopsis overexpressing *CsLAC4*. **A** Schematic of the construction of stably transformed *Arabidopsis thaliana*. **B** Semiquantitative results of *CsLAC4* in different lines of *Arabidopsis*. **C** Identification of the T2 generation of homozygous Arabidopsis was performed on 1/2 MS medium with kanamycin.


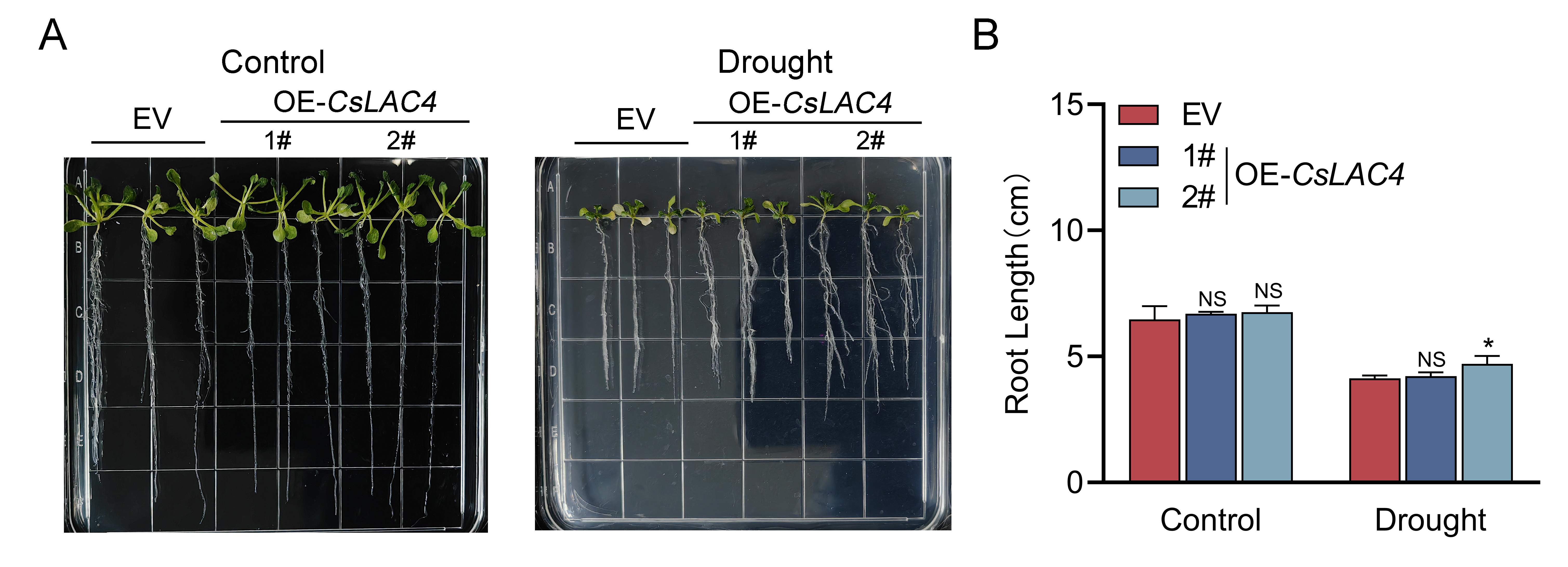


**Fig. S2** Validation of drought tolerance in OE-*CsLAC4* Arabidopsis. **A** Phenotypes of transgenic Arabidopsis grown under drought conditions. **B** Root length of transgenic Arabidopsis under normal conditions or drought trestment (n=3). Each bar indicates the mean ± SD. Asterisks indicate significant differences relative to EV, **p* < 0.05.


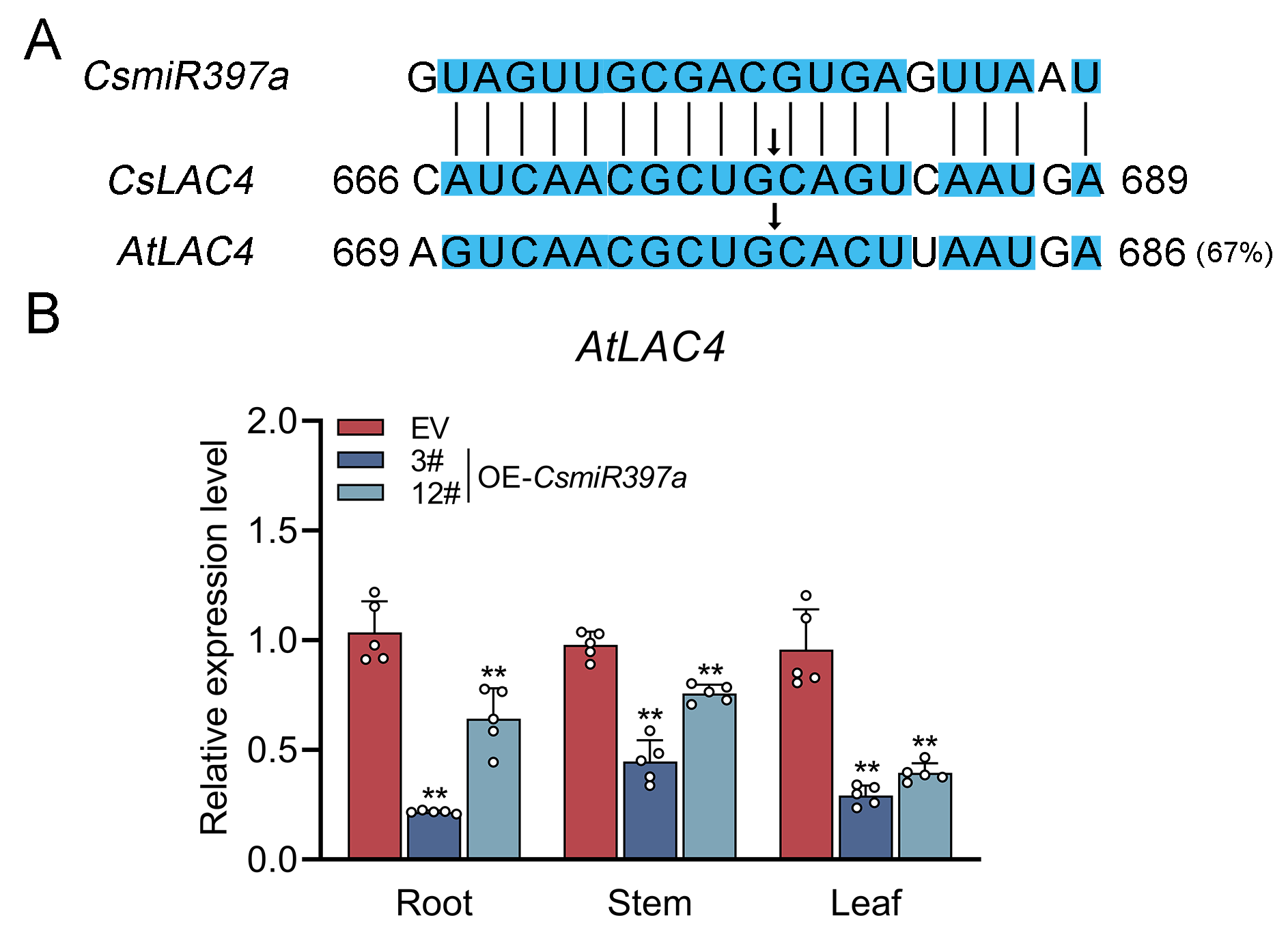


**Fig. S3** Validation of the regulation of *AtLAC4* by *CsmiR397a* in Arabidopsis. **A** Similarity analysis of the *CsmiR397a* cleavage site of *CsLAC4* and *AtLAC4*. **B** Expression levels of *AtLAC4* in different tissues of OE-*CsmiR397a* *Arabidopsis thaliana* (n=5). Each bar indicates the mean ± SD. Asterisks indicate significant differences relative to EV, ***p* < 0.01.


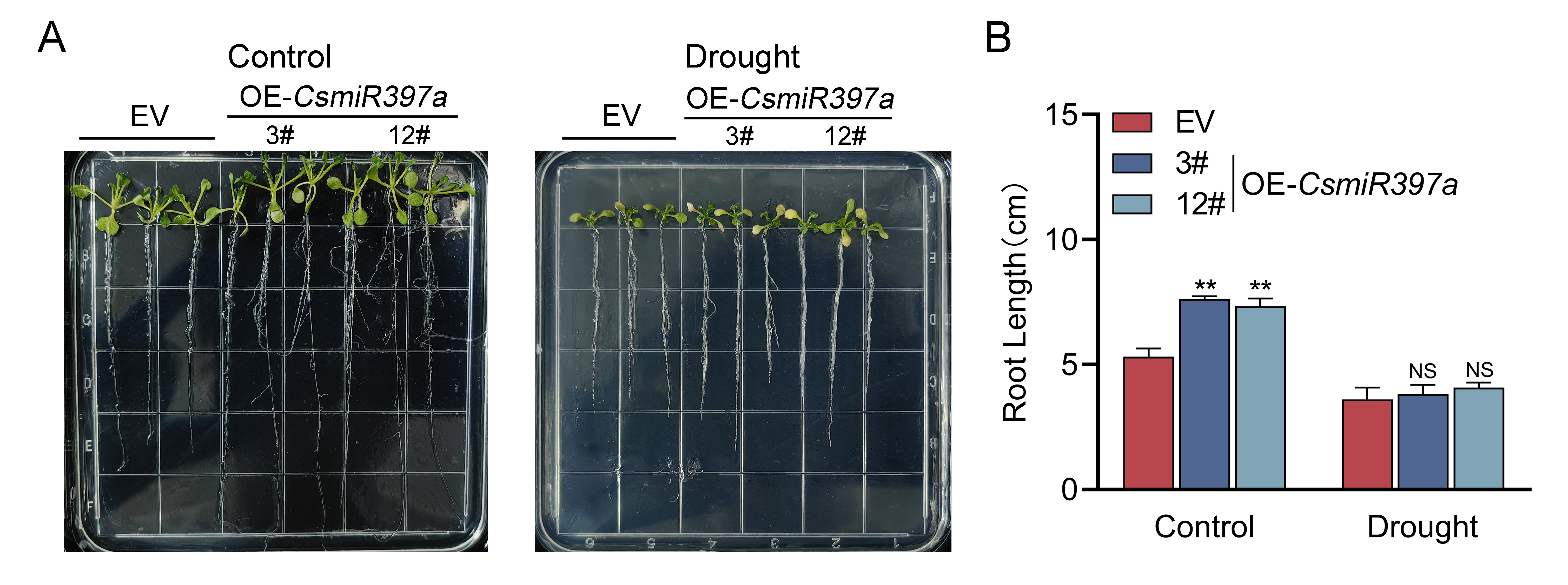


**Fig. S4** Validation of drought tolerance in OE-*CsmiR397a* Arabidopsis. **A** Phenotypes of transgenic Arabidopsis grown under drought conditions. **B** Root length of transgenic Arabidopsis under normal conditions or drought trestment (n=3). Each bar indicates the mean ± SD. Asterisks indicate significant differences relative to EV, **p* < 0.05.


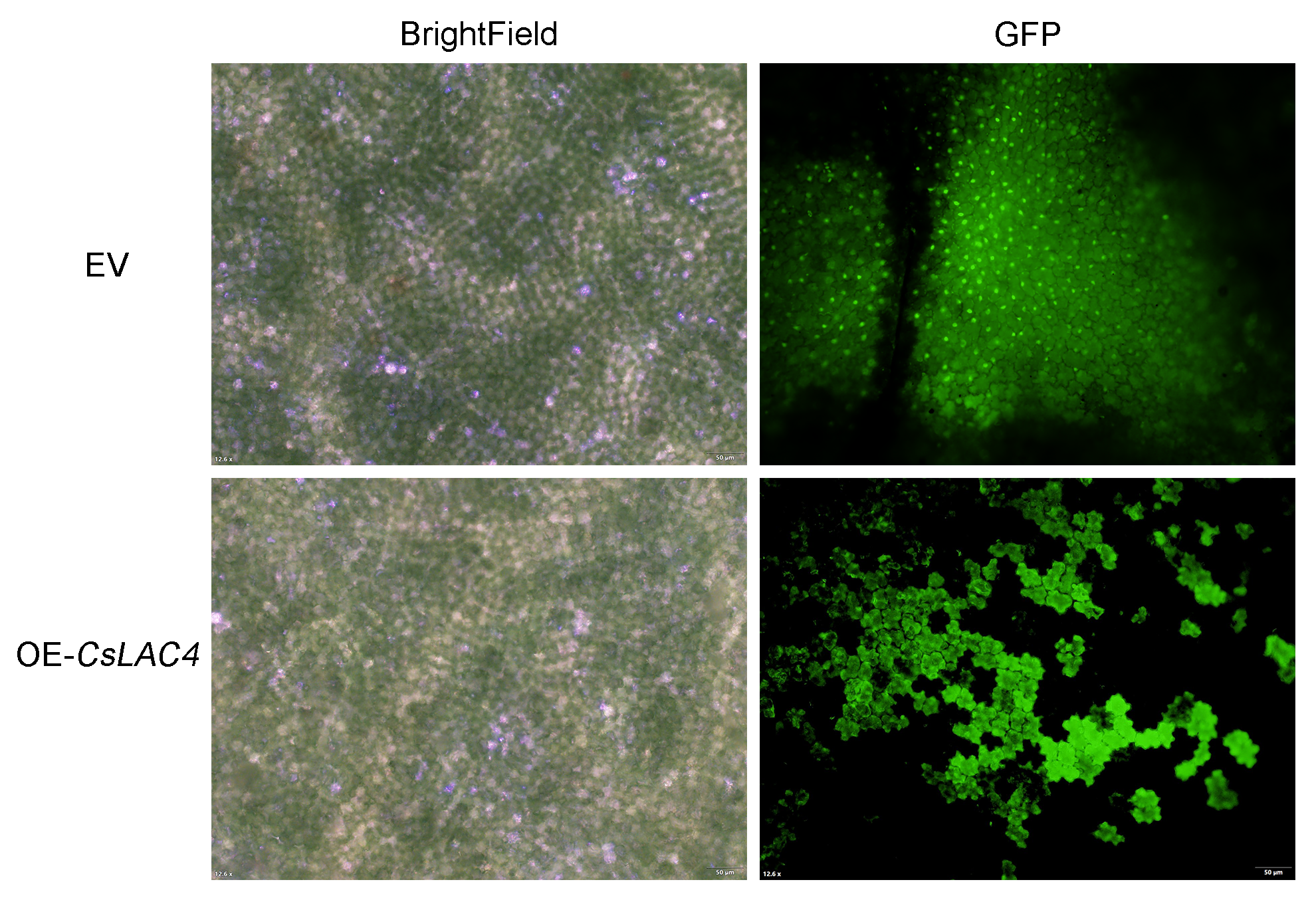


**Fig. S5** EV, CsLAC4-GFP fusion protein in bright field and GFP field of fluorescence microscope.
