# Supplementary material for: CsLAC4, regulated by CsmiR397a, confers drought tolerance to the tea plant by enhancing lignin biosynthesis
Source: Stress Biol. 2024 Dec 6;4(1):50. doi: 10.1007/s44154-024-00199-1 (PMC11624182; doi:10.1007/s44154-024-00199-1)
Supplement: Supplementary file 2 — Additional file 2: Table S1 Primers used in this study. [file 44154_2024_199_MOESM2_ESM.docx]

**Table S1** Primers used in this work

| **Name** | **Primer** | **Sequence (5’-3’)** | **Function** |
| --- | --- | --- | --- |
| *CsLAC4*  (CSS0015036) | Forward | ATGGCATACTATCGGATTCAGGCAGC | Gene cloning |
|  | Reverse | CTAACACATTGGAAGGTCACTTGGAG |  |
| *CsLAC4*  (CSS0015036) | Forward | GTTCAGTGTGGTGTTGAAGAATG | qRT-PCR |
|  | Reverse | CCATCTGACCATCCTGTTCTTAG |  |
| *AtLAC4*  (AT2G38080) | Forward | GGGTCTCATATGGTTTGGTTTCT |  |
|  | Reverse | GTGGATGGAGACGTTGTACTTG |  |
| *CsActin* | Forward | TAGAAACCCCAAGTACCCTCG |  |
|  | Reverse | TGCTTTCTTCGTCCCATCAG |  |
| *AtActin* | Forward | GTCGTACAACCGGTATTGTGCT |  |
|  | Reverse | TGTCTCTTACAATTTCCCGCTCT |  |
| *AtU6* | Forward | CGGGGACATCCGATAAAATT |  |
|  | Reverse | TTGGACCATTTCTCGATTTG |  |
| *CsmiR397a* | Forward | TAATTGAGTGCAGCGTTGATG |  |
|  | Reverse | provide by kit (Accurate, China) |  |
| *CsmiR222* | Forward | TTTCCAAGACCACCCATGCCGA |  |
|  | Reverse | provide by kit (Accurate, China) |  |
| *35S*::*CsLAC4-LUC* | Forward | TTTGGAGAGAACACGAAGCTTATGGCATACTATCGGATTCAGGC | Dual luciferase assay |
|  | Reverse | CGCTCTAGAACTAGTGGATCCACACATTGGAAGGTCACTTGGAG |  |
| *35S*::*mCsLAC4-LUC* | Forward | TTTGGAGAGAACACGAAGCTTATGGCATACTATCGGATTCAGGC |  |
|  | Reverse | CGCTCTAGAACTAGTGGATCCACACATTGGAAGGTCACTTGGAG |  |
| *35S*::*CsLAC4-GFP* | Forward | TCCTCCTCCTCTAGAGGATCCATGGCATACTATCGGATTCAGGC | Subcellular Localization  analysis |
|  | Reverse | ATTTGGAGAGGACAGGGTACCACACATTGGAAGGTCACTTGGAG |  |
| *CsmiR397a-*agomir | sense: UAAUUGAGUGCAGCGUUGAUG  antisense: UCAACGCUGCACUCAAUUAUU | | miRNA overexpression |
| Negative control | UCACAACCUCCUAGAAAGAGUAGA | |  |
